# The intention to use mHealth applications among Dutch older adults prior and during the COVID pandemic

**DOI:** 10.3389/fpubh.2023.1130570

**Published:** 2023-06-13

**Authors:** Floris Ruben Tobias van Elburg, Joris van de Klundert, Anna Petra Nieboer, Marjan Askari

**Affiliations:** ^1^Erasmus School of Health Policy & Management, Erasmus University Rotterdam, Rotterdam, Netherlands; ^2^School of Business, Universidad Adolfo Ibanez, Santiago, Chile; ^3^Rotterdam School of Management, Erasmus University, Rotterdam, Netherlands

**Keywords:** technology acceptance model, intention to use, older adults, medical applications, mHealth, COVID-19, pandemic

## Abstract

**Background:**

Mobile health (mHealth) applications are widely valued for their potential to increase self-management among older adults and reduce their healthcare demands. However, the intention to use mHealth of Dutch older adults before the COVID-19 pandemic was modest. Healthcare access was considerably reduced during the pandemic and mHealth services substituted for in person health services. As older adults utilize health services more frequently and have been particularly vulnerable to the pandemic, they can be viewed to have especially benefitted from the transition toward mHealth services. Furthermore, one might expect their intention to use these services and reap the potential benefits has increased, especially during the pandemic.

**Objective:**

The aim of this study was to examine whether the intention of Dutch older adults to use medical applications increased during the COVID pandemic and how the explanatory power of the extended Technology Acceptance Model (TAM) developed for this purpose was affected by the onset of the pandemic.

**Methods:**

We conducted a cross-sectional survey using two samples collected before (*n* = 315) and after (*n* = 501) the onset of the pandemic. Data was collected using questionnaires which were distributed digitally and on paper, by convenience sampling and snowballing. Participants were 65 years or older, lived independently or in a senior living facility, without cognitive impairment. A controlled analysis was performed to test for significant differences in the intention to use mHealth. The before and after differences in extended TAM variables and their relationship with intention to use (ITU) were analyzed using controlled (multivariate) logistic and linear regression models. These models were also used to explore whether the onset of the pandemic had an effect on ITU not captured by the extended TAM model.

**Results:**

While the two samples differed in ITU (*p* = 0.017; uncontrolled) there was no statistically significant difference in ITU in the controlled logistic regression analysis (*p* = 0.107). The scores of the extended TAM variables explaining intention to use were all significantly higher, except for Subjective norm and Feelings of Anxiety. The relationships of these variables with intention to use before and after the onset of the pandemic were similar, except for Social relationships which lost its significance. We found no indications of effects of the pandemic on intention to use not captured by our instrument.

**Conclusion:**

The intention to use mHealth applications of Dutch older adults has not changed since the onset of the pandemic. The extended TAM model has robustly explained intention to use, with only minor differences after the first months of the pandemic. Interventions targeting facilitation and support are likely to promote the uptake of mHealth. Follow-up studies are needed to investigate whether the pandemic has had long term effects on the ITU of the older adult.

## Introduction

Since the onset of the COVID-19 pandemic, governments across the globe have imposed mandatory limitations in mobility and many citizens have adopted additional voluntary limitations (1, 2). The latter holds especially true for older adults, who have been identified by the World Health Organization as being significantly more at risk to experience negative health effects, including increased mortality risk, from COVID-19 (3–7). In fact, many of the COVID-measures taken by governments especially aimed to protect older adults (1, 4). Together with access restrictions imposed by healthcare providers, these limitations have resulted in huge reductions in delivery of in person healthcare services globally (8–10). Thus, the pandemic has especially and negatively impacted older adults in particular, due to the relatively high morbidity and mortality risks and because of their more frequent usage of healthcare services (4, 11–16).

Hence, the COVID-19 pandemic necessitated older adults to resort to alternative health service modalities which reduced health risks and met the mobility limitation measures. The pandemic caused a tremendous increase in the uptake of digital health technologies (17–20). Mobile health, or mHealth, are mobile applications that run on smartphones or tablets and can gather health information, monitor health and support activities regarding an individual’s health (21–24). A previous study indicated that mHealth could become a vital part of innovation in the patient-physician relationship (25). Current literature also shows that mHealth can have positive health effects for the older adult. For example, mHealth technology has proven effective for the management of chronic diseases (13), which can improve an individual’s overall health along with quality of life. Furthermore, mHealth enables remote health service delivery and has been shown to improve self-reliance, health behavior and medication adherence of older adults (13, 26–28), possibly reducing the demand for hospital services (21, 22, 29, 30). A systematic review has shown that mHealth has been successfully deployed to promote health services during the COVID-19 pandemic (4). Another systematic review identified mHealth as a very suitable alternative to patient-physician contacts during the COVID-19 pandemic (31).

Older adults have been identified as the subpopulation that can benefit most from mHealth services (27, 32, 33). Despite the evidence of effectiveness and potential, older adults have often been reluctant to adopt mHealth (34–39). Prior to the pandemic, half of the Dutch older adults had no intention to use mHealth applications (37–39). One might hypothesize that the extraordinary circumstances of the pandemic increased the need for digital healthcare applications and therefore increased the intention to use such applications among the older adult. Our first research question is therefore whether the extraordinary circumstances of the pandemic, which severely limited the access to usual in-person health services, have increased the intention to use mHealth among older adults (RQ1).

The uptake of mHealth by older adults has been studied previously on the basis of the Technology Acceptance Model (TAM), which posits that actual use is largely determined by the intention to use (ITU) (40). TAM has been studied widely, also in relation to mobile applications and in the health services domain. Furthermore, several studies have focused on health technology acceptance by older adults (37–39, 41–44). Even though TAM in its original form can have a high explained variance in technology adoption (45), recommendations have been made to integrate additional variables to improve the context specificity (13, 42–47). This has led to the inclusion of additional factors in TAM2 (48), the Senior Technology Acceptance Model (44) and the expanded TAM version of Wu (49). Following these extensions, an earlier study on the ITU of mHealth among Dutch older adults presented and validated an extended TAM model which identified Perceived usefulness, Perceived ease of use, Attitude toward use, Subjective norm, Sense of control, Feelings of Anxiety, Personal innovativeness, Social relationships, Self-perceived effectiveness and Service availability as significant factors (39).

One may question whether the factors identified in this (tailored) TAM model and the associations between these factors and ITU have remained valid for older adults during the pandemic. This question is especially relevant because of the aforementioned importance of access to alternatives for in person health services during a pandemic for older adults. Understanding the factors influencing the intention to use mHealth during a pandemic, and the associations between these factors and intention to use, can help tailor mHealth solutions, corresponding health services, and policy measures. Hence, our second, composed, research question studies whether:

- The extended TAM factors explaining ITU have been perceived differently after the onset of the pandemic (RQ2a).
- The relationships between the extended TAM factors and ITU have changed after the onset of the pandemic (RQ2b).
- The pandemic has had a significant effect on ITU not captured by the factors included in the extended TAM model (RQ2c).

Therefore, the aim of this study was to investigate whether the intention to use mHealth applications of Dutch older adults has changed after the onset of the COVID-19 pandemic. In addition, we sought to investigate whether the relationships between the extended TAM factors and ITU have changed since the onset of the pandemic.

The paper first describes the methods for data collection and statistical analysis used to answer each research question. The results section shows the results of statistical analysis per research question. The discussion reflects on principal findings and compares the results to findings from previous studies. The manuscript ends with limitations and conclusions.

## Methods

### Study design and data collection

A cross-sectional study was designed comparing two samples of data to investigate the intention to use medical apps before and during the first COVID pandemic wave. Data were collected in the regions of Utrecht, Zuid-Holland, Noord-Holland and Brabant in the Netherlands by distributing a questionnaire among Dutch older adults. The first data set was gathered during the first 6 months of 2019 and the second one between February and June 2020. This approach provided one large data set consisting of two samples, one before and one after the onset of the COVID pandemic in early 2020. Our cohort consisted of 816 older adults: 315 older adults in the ‘pre-COVID’ group and 501 in the ‘during-COVID group’. Within the manuscript, the sample collected after the onset of the pandemic is also referred to as the ‘during-COVID group’. The inclusion criteria were: (1) seniors aged 65 years and older, (2) who lived independently or in a senior living facility, and (3) without cognitive impairment. Data collection of both samples followed the same methods. The 2019 data collection processes have been described extensively in Askari et al. (39), which also presents an analysis of factors associated with ITU pre-COVID-19. Below, we refer to the methods described when possible, while providing brief explanations and describing additional methods as necessary for this manuscript to be self-contained.

The questionnaire used in this study was composed of items from various well-validated measuring instruments (39). It was subsequently checked for quality by five experts (three eHealth experts, one geriatric nurse and one physician). Additionally, a group of four older adults gave feedback to improve structure and readability. The questionnaires were distributed both digitally and on paper and respondents who preferred the paper version were offered help to administer the questionnaires (39). Distribution of questionnaires followed the recruitment techniques of convenience sampling and snowball sampling. The mobility restrictions that followed the onset of the pandemic severely limited deployment of the paper version for the second round of data collection from February to June 2020. As a result, the majority of the data for this group was gathered through online questionnaires. The Checklist for Reporting Results of Internet E-Surveys (CHERRIES) was used for the reporting of the online questionnaire (see Multimedia Appendix 1) (50).

An informed consent form was signed by all the participants before participation and the data was pseudonymized to ensure anonymity. Our study was approved by the Medical Ethical Commission of the Erasmus Medical Center under the number MEC-2018-120.

### Technology acceptance model

The determinants for mHealth use that are used in this study predominantly originate from the Technology Acceptance Model (TAM) (40). Intention to use is the outcome variable of interest in TAM. ITU was measured by submitting three statements to the respondent using a 5-point Likert scale (1 = completely disagree, 2 = disagree, 3 = neutral, 4 = agree, 5 = completely agree). ITU was transformed into a binary variable to enhance interpretability of results. As independent variables, we included the factors from the extended TAM model used and validated by Askari et al. (39), as described in the introduction. As is the case for the outcome variable, the questionnaire includes multiple statements for each explanatory TAM factor, which are answered on a five-point Likert scale. The factors included were: Perceived ease of use (4 statements, e.g., ‘It is easy to use medical applications for remote care’), Perceived usefulness (3 statements, e.g., ‘I find it useful to use medical applications for remote care’), Attitude toward use (4 statements, e.g., ‘Using medical applications for remote care would be a good idea’) and Subjective norm (3 statements, e.g., ‘People who are important to me think I should use medical applications for remote care’) together with factors specific for older adults, i.e., Sense of control (2 statements, e.g., ‘I have resources, knowledge and ability to use medical applications for remote care’), Anxiety toward use (2 statements, e.g., ‘I hesitate to use medical applications for remote care for fear of making mistakes that I cannot correct’), Personal innovativeness (4 statements, e.g., ‘I like to experiment with new information technology’), Social relationships (3 statements, e.g., ‘I am satisfied with the support of my family and friends’), Self-perceived effectiveness of use (2 statements, e.g., ‘I could perform a task on a medical application if I only have the manual’), Service availability (3 statements, e.g., ‘I have the ability to use medical applications for remote care anytime, anywhere’) and Facilitating circumstances (5 statements, e.g., ‘I have the knowledge needed to use medical applications’). Multimedia Appendix 2 provides descriptions of each of the factors included. For each TAM variable a factor score was generated by calculating the average score of the corresponding statements. Responses with missing values on any of the TAM variables or control variables were listwise deleted.

### Statistical analyzes

Descriptive statistics of the samples were calculated for the entire study population, as well as separately for the groups of which data was collected before and during the pandemic. For continuous variables, the mean and standard deviations (SD) were calculated and for categorical variables, percentages were used. The characteristics of the two samples, including their ITU, were compared using the Chi-Square test for nominal variables and the Mann–Whitney *U* test for continuous variables.

To answer the first research question (RQ1) whether the extraordinary circumstances of the pandemic increased the intention to use mHealth among older adults, we conducted a Chi squared test for differences between the pre-pandemic sample and the second sample. Next, we performed logistical regression analysis with ITU as the dependent variable: first performing univariate and then multivariate analysis. Multivariate regression analysis was done while controlling for demographic variables based on expert opinion and those that were significantly associated with ITU in a univariate regression of the complete sample: age, sex, education and Assessment of Activities of Daily Living, Self-Care, and Independence (ADL). The ADL score (51) measures the extent to which the respondent needs help with 16 daily activities, with a high score indicating greater ability to autonomously perform activities of daily living (51–54). An overview of univariate analysis of the candidate control variables on ITU can be found in Multimedia Appendix 3. The control variables were tested for multicollinearity using a correlation matrix. As none of the control variables had a correlation larger than 0.5 or lower than −0.5, there were no limitations to jointly include them in the multivariate analyzes (55).

To answer the second research question, which was to determine which factors influence the intention to use mHealth, we first computed average scores of the TAM factors included as explanatory variables of ITU. We then analyzed whether these scores differed significantly between the two samples using independent samples t-tests to answer RQ2a. To provide a stronger answer to RQ2a and reveal differences in the values of the explanatory TAM variables before and after the onset of the pandemic we additionally conducted linear regression analysis with the pandemic (0 = before onset/1 = after onset) and the controls as described above as independent variables and each of the TAM factor scores as the dependent variable.

To answer RQ2b and investigate the relationship between the explanatory TAM variables and ITU, the generated factor scores of the technology acceptance factors were used as input for controlled logistic regression analysis. For each explanatory factor we conducted logistic regressions including the TAM factors and selected controls as independent and ITU as dependent variable for the pre-COVID and during COVID samples separately. The odds ratio (OR), coefficient (β) together with *p*-value and standard error (SE) for each factor is reported.

To deepen our answer to RQ2b, an additional assessment of differences between the two samples was conducted using pooled models for each of the explanatory variables (with all data from both samples) which included an interaction term for each TAM factor and a logistic variable distinguishing the two samples (0 = before onset/1 = after onset). For these models we also report the *p*-values, standard error and the coefficients (β) of the explanatory variable and the interaction term.

Lastly, we compared the results of two logistic regression models, respectively with and without a logistic variable distinguishing the samples, and including all TAM variables and controls and compared explained variance to answer the question whether the pandemic had effects on ITU not captured by the extended TAM model (RQ2c).

All the statistical analyzes were performed using SPSS Statistics (IBM Corporation, version 25).

## Results

### Population characteristics

In total, 816 respondents were included in the study. Our cohort had an average age of 74 years (SD = 6.3 years). Out of the 816 participants, 48.8% were male. While the vast majority of the study sample had prior experience with the internet (92.9%), only 186 respondents (22.8%) had used a medical app before. More than half of the studied population (62.3%) had the intention to use a medical application. A summary of the population characteristics can be found in Table 1.

**Table 1:** Description of the sample and population characteristics in the total population and per group.

Characteristics	Total population (816)	Pre-COVID (*N* = 315; 38.6%)	During COVID (*N* = 501; 61.4%)	*P-*value
Age in years, mean (sd)	74.0 (6.3)	74.4 (7.0)	73.8 (5.8)	0.797^b^
Sex (male), no. (%)	392 (48.0)	144 (45.7)	248 (49.5)	0.240^a^
Education, no. (%)				0.662^a^
Secondary education	126 (15.4)	48 (15.2)	78 (15.6)	
Post-secondary or graduate education	387 (47.4)	140 (44.4)	247 (49.3)	
Post graduate education	288 (35.3)	114 (36.2)	174 (34.7)	
Marital status, no. (%)				0.001^a^
Wedded	463 (56.7)	167 (53.0)	296 (59.1)	
Divorced	78 (9.6)	47 (14.9)	31 (6.2)	
Widowed	185 (22.7)	73 (23.2)	112 (22.4)	
Unwedded	45 (5.5)	17 (5.4)	28 (5.6)	
Sustainable cohabitation	26 (3.2)	7 (2.2)	19 (3.8)	
Living arrangement, no. (%)				0.001^a^
Living independently, alone	259 (31.7)	108 (34.3)	151 (30.1)	
Living independently, with other	430 (52.7)	143 (45.4)	287 (57.3)	
Senior living facility, alone	52 (6.4)	30 (9.5)	22 (4.4)	
Senior living facility, with other	59 (7.2)	29 (9.2)	30 (6.0)	
Adl score, mean (sd)	15.0 (1.8)	14.7 (2.3)	15.1 (1.4)	<0.282^b^
Prior experience with internet, no. (%)	757 (92.8)	271 (86.0)	486 (97.0)	<0.001^a^
Prior experience with medical apps, no. (%)	186 (22.8)	53 (16.8)	133 (26.5)	0.001^a^
Online questionnaire, no. (%)	588 (72.1)	114 (36.2)	474 (94.6)	<0.001^a^
Intention to use mobile medical apps, no. (%)	508 (62.3)	180 (57.1)	328 (65.5)	0.017^a^

a Chi-Squared test.

b Mann–Whitney *U* test.

Table 1 shows lower prior experience with the internet (86.3% vs. 97.0%) and lower prior experience with medical applications (16.9% vs. 26.5%) for the pre pandemic sample. This might be explained by the limitation to recruit respondents only capable of responding on paper during the pandemic. The proportion of questionnaires that was filled in digitally versus on paper differed significantly between the two groups (*p* = <0.001). In 2019, one-third of the population (36.2%) submitted their answers digitally, while in 2020 almost all questionnaires were submitted online (94.6%).

While the implications of these differences in response modality will be discussed in the discussion section, we already note that they may be taken into account when observing the uncontrolled result for the main variable of interest, intention to use. In 2019, 57.1% of the respondents indicated the intention to use a medical application and this percentage increased significantly to 65.5% in 2020 (*p* = 0.017).

### Regression analysis results

The first research question of this study addressed whether the unique circumstances of the pandemic have led to a difference in intention to use mHealth among older adults. We hypothesized that the ITU of Dutch older adult had increased after the onset of the COVID pandemic. Table 1 presents the data describing the two samples and reveals the two samples differ significantly on several technology use related factors and in particular on the intention to use mHealth (*p* = 0.017). The results of regression analysis are shown in Table 2. The result of the uncontrolled logistic regression suggests that the ITU may differ between the two groups (*p* = 0.017). However, controlling for previously identified covariates, there is no significant difference in ITU between the two samples (*p* = 0.107), which answers the first research question (RQ1).

**Table 2:** Difference in intention to use between pre-COVID and during COVID groups.

	*P-*value	OR (CI 95%)	β (SE)
Uncontrolled	0.017	1.42 (1.07–1.90)	0.35 (0.148)
Controlled^a^	0.107	1.29 (0.95–1.76)	0.26 (0.158)

^a^Controlled for age, sex, education level, ADL score.

The second research question was composed of three sub-questions and looked into the explanation of the ITU through TAM factors. First, we investigated whether the explaining TAM factors have been perceived differently after the onset of the pandemic (RQ2a). The results of the controlled linear regression analyzes are shown in Table 3 and shed further light on differences in the TAM variable values before and after the onset of the pandemic. The change in factor scores is shown as a coefficient of the pandemic (0 = before onset/1 = after onset). It shows that all explanatory TAM variables scored significantly higher after the onset of the pandemic, except for Subjective norm and Feelings of anxiety. The uncontrolled average scores for each of the explanatory TAM variables in both groups can be found in Multimedia Appendix 4.

**Table 3:** TAM factor differences between pre-COVID and during COVID groups.

TAM factor^a^	*β* (CI 95%)	SE	*P-*value
Perceived usefulness	0.20 (0.09–0.32)	0.060	0.001
Perceived ease of use	0.17 (0.06–0.29)	0.058	0.003
Attitude toward use	0.21 (0.09–0.33)	0.062	0.001
Subjective norm	0.10 (−0.05–0.24)	0.072	0.183
Sense of control	0.23 (0.10–0.37)	0.069	0.001
Feelings of anxiety	−0.07 (−0.20–0.07)	0.068	0.345
Personal innovativeness	0.16 (0.02–0.30)	0.070	0.022
Social relationships	0.11 (0.02–0.20)	0.045	0.019
Self-effectiveness	0.14 (0.02–0.26)	0.061	0.027
Service availability	0.14 (0.03–0.26)	0.060	0.018
Facilitating circumstances	0.10 (0.01–0.19)	0.045	0.033

^a^Controlled for age, sex, education level, ADL score.

Second, to study whether relationships between the explanatory TAM variables and ITU had changed since the onset of the pandemic, controlled logistic regression analysis was done (RQ2b). Table 4 provides an overview of the results for controlled logistic regression analyzes for the pre- and during COVID groups separately. All explanatory variables showed significant relationships in the pre-Covid group. Social relationships was the only variable that did not have a significant relationship with ITU in the during COVID group. All variables that were significantly associated with ITU in both samples showed higher odds ratios with ITU in the during COVID group, except for Self-perceived effectiveness.

**Table 4:** Logistic regression of TAM-variables and their relationship with intention to use.

Clusters	Pre-COVID^a^				During COVID^a^			
	OR (95%CI)	*P-*value	*β*	S.E.	OR (95%CI)	*P-*value	*β*	S.E.
Perceived usefulness	4.87 (3.19–7.44)	<0.001	1.58	0.216	8.42 (5.40–13.12)	<0.001	2.13	0.226
Perceived ease of use	3.74 (2.50–5.58)	<0.001	1.32	0.204	4.06 (2.82–5.87)	<0.001	1.40	0.187
Attitude toward use	9.09 (5.23–15.79)	<0.001	2.21	0.282	10.73 (6.76–17.04)	<0.001	2.37	0.236
Subjective norm	1.44 (1.12–1.86)	0.005	0.37	0.130	1.50 (1.21–1.85)	<0.001	0.40	0.108
Sense of control	3.27 (2.35–4.54)	<0.001	1.18	0.167	3.70 (2.74–4.98)	<0.001	1.31	0.152
Feelings of anxiety	0.66 (0.51–0.87)	0.003	−0.41	0.136	0.49 (0.39–0.62)	<0.001	−0.71	0.116
Personal innovativeness	2.06 (1.55–2.73)	<0.001	0.72	0.144	2.07 (1.65–2.60)	<0.001	0.73	0.116
Social relationships	1.65 (1.06–2.55)	0.026	0.50	0.224	1.09 (0.80–1.49)	0.571	0.09	0.157
Self-perceived effectiveness	2.61 (1.85–3.66)	<0.001	0.96	0.174	2.30 (1.76–3.01)	<0.001	0.83	0.138
Service availability	3.40 (2.31–5.02)	<0.001	1.22	0.198	3.69 (2.65–5.12)	<0.001	1.31	0.168
Facilitating circumstances	3.54 (2.22–5.64)	<0.001	1.26	0.237	3.59 (2.43–5.32)	<0.001	1.28	0.200

^a^Controlled for: age, sex, education level, ADL score.

Table 5 shows results of multivariate logistic analysis with interaction terms per acceptance factor. The results from Table 4 indicate that the relationships of the explanatory TAM variables, with the exception of Social relationships, with ITU were stronger after the onset of the pandemic. However, the insignificance of the interaction terms of the pooled analyzes as shown in Table 5 indicate that these relationships have not been significantly impacted by the pandemic.

**Table 5:** Interaction terms of TAM-variables and their relationship with intention to use.

Clusters	During COVID vs. pre-COVID^a^			
	OR (95%CI)	*P-*value	*β*	S.E.
Perceived usefulness	1.67 (0.92–3.06)	0.094	0.52	0.307
Perceived ease of use	1.07 (0.63–1.82)	0.810	0.07	0.271
Attitude toward use	1.21 (0.60–2.46)	0.599	0.19	0.362
Subjective norm	1.05 (0.76–1.45)	0.781	0.05	0.165
Sense of control	1.09 (0.71–1.68)	0.695	0.09	0.219
Feelings of anxiety	0.79 (0.57–1.10)	0.160	−0.24	0.169
Personal innovativeness	1.00 (0.70–1.42)	0.976	−0.01	0.181
Social relationships	0.69 (0.41–1.17)	0.164	−0.37	0.268
Self-perceived effectiveness	0.88 (0.58–1.34)	0.552	−0.13	0.214
Service availability	1.04 (0.63–1.72)	0.870	0.04	0.256
Facilitating circumstances	0.99 (0.54–1.80)	0.966	−0.01	0.307

^a^Controlled for: age, sex, education level, ADL score.

The third and final sub-question of our second research question looked into any effects of the pandemic on ITU that were not captured by the explanatory TAM variables (RQ2c). Table 6 shows the explained variance of the complete model, including all explanatory TAM variables and controls. Explained variance of the model was calculated for the total study population, respectively, with and without a logistic variable distinguishing the samples of pre-COVID and during COVID groups. The explained variance of the model was marginally higher in the pre-COVID group, indicating the model performed slightly better before the onset of the pandemic. However, addition of the logistic variable to the pooled model did not change the explained variance, indicating that there were no effects of the pandemic on ITU not already captured by the extended TAM model.

**Table 6:** Explained variance of intention to use of the model with all control variables and all TAM variables.

	Cox and Snell *R*^2^	Nagelkerke *R*^2^
Pooled model without distinguishing variable	0.419	0.572
Pooled model with distinguishing variable	0.419	0.572
Pre-COVID group	0.462	0.621
During COVID group	0.402	0.556

## Discussion

### Principal results

Our first research question asked whether the intention to use mHealth of Dutch older adults increased after the onset of the pandemic. While an initial uncontrolled analysis suggests that the ITU of Dutch older adult may have increased – based on the result of the Chi2 test (*p* = 0.017) and a univariate regression analysis (*p* = 0.017) – we found no evidence of differences in ITU before and after the onset of the pandemic, after controlling for socio-demographic factors (*p* = 0.107). Second, we studied changes in the determinants of the intention to use and their relationship with intention to use to explain (possible changes in) intention to use. After controlling for demographics, Social relationships was the only explanatory variable that did not remain significant after the onset of the pandemic. Except for Self-perceived effectiveness, all explanatory TAM variables showed larger OR’s after onset of the pandemic. However, regression analysis with interaction terms showed that the relationships between these variables and ITU were not significantly affected by the pandemic. In other words, the quantitative relationships identified by the extended TAM model appeared robust even during the unusual circumstances that particularly affected older adults. As the ITU itself was not significantly affected when controlled for demographics (or only mildly in uncontrolled analysis) this brings forth the question whether the unusual circumstances of the pandemic have affected the ITU in ways that are not captured by other extended TAM variables. Our final results (Table 6) indicate that this is not the case. Altogether, the results confirm the validity and completeness of the extended TAM model for older adults in The Netherlands, even during pandemic circumstances.

The results were obtained using a dichotomous definition of intention to use, which is originally measured on a five-point Likert scale. The dichotomous modeling of intention to use is based on the close relationship between intention to use and actual use, which ultimately is of a dichotomous nature. The use of a dichotomous model of intention to use aligns with previous research on this subject (37–39). However, the dichotomous model can be perceived as crude, especially as intention to use is measured using a five point Likert scale. A more sensitive model in which intention to use is modeled using a continuous variable over the five point scale finds that the controlled difference in intention to use after the onset of the pandemic is significant (*β* = 0.14, *p* = 0.047). This was investigated using linear regression with a continuous definition of ITU and results are displayed in Multimedia Appendix 5. This model could be valuable, for instance because it may pick up policy effects earlier. Future studies could consider using this more sensitive model in addition to models with dichotomous ITU definitions.

The results reflect on a period with severe limitations in access to care as physical visits were restricted to a minimum. Moreover, older adults were known to be especially vulnerable for COVID-19 and had reasons to associate hospital visits with increased risks of obtaining COVID-19. Our findings suggest that even when the circumstances changed and strongly favored the use of mHealth, the intentions of older adults to use these technologies appears to have remained unaltered (or that the alterations have been minor at best). This reinforces previous findings that older adults are reluctant to take up mHealth (30–35). Subsequently, it confirms the need for targeted and dedicated policies for older adults especially during challenging circumstances such as the pandemic.

To the best of our knowledge, this study is the first to investigate differences in ITU and its determinants between comparable samples before and after the onset of the pandemic. Hence, it provides novel evidence on the intention to use mHealth during the pandemic and how to significantly improve it. Our findings strongly suggest that any policy measures to improve mHealth uptake among the older adult should target the well-known determinants of the extended TAM model shown to be significant in Table 4. Further research on how to impact these variables is merited.

### Comparison with prior work

Much research has appeared on a variety of mHealth interventions and the determinants of their use by older adults after the onset of the COVID-19 pandemic (3, 19, 56–58). The literature generally reports that mHealth can play a vital role for access and continuity of care during the pandemic and that implementation of mHealth solutions is recommended (7, 19, 59–61).

Uptake of mHealth by older adults has been reported to have remained a challenge during the pandemic and largely depends on the support that is available to them (4, 56, 60). This has led to the recommendation that the use of mHealth by older adults has to be facilitated by family and friends (4). While our findings confirm that the intention to use mHealth has not (or hardly) changed during the pandemic they suggest that interventions exploiting their social relationships are unlikely to be effective. We find that social relationships became insignificant as a factor explaining ITU after the onset of the pandemic, as is also confirmed in another study (3). Additionally, the importance of facilitation and support by governmental bodies and other parties, such as healthcare providers, is reported in several studies (4, 56, 60). These results are consistent with our main finding that the intention to use mHealth of older adults themselves has not or barely increased during the pandemic, despite its potential to address their specific demands. Thus, rather than relying on the intrinsic motivation of older adults, targeted policies improving support and facilitation appear needed to reap the benefits of mHealth uptake as especially valuable during the pandemic. Our findings therefore suggest that future research and policies to promote mHealth during pandemic circumstances should not focus on family and friends but rather target other supporting parties of the older adult population. For example, healthcare professionals with a guiding role like nursing specialists or physical therapists could play a role in the promotion of these technological applications (4, 56, 60).

## Limitations

To investigate potential differences between TAM factors and their relationships with ITU following the pandemic, we collected data from two different samples before and after the onset of the pandemic, respectively. A methodological limitation of this study design is that we can only compare the two samples but are unable to study longitudinal individual level effects of the pandemic on ITU. Future studies are needed to investigate the long-term exposure to the pandemic circumstances and the effect on ITU.

A second limitation stems from the data collection challenges during the pandemic. This generated a large increase in the percentage of questionnaires completed online. As a result, differences in the prior experience with medical apps and the intention to use were found. These differences are displayed in Table 1. The difference in ratio paper/online questionnaires between the two groups correlates with the pandemic. As the number of paper based questionnaires received was small during the pandemic, it has not been possible to correct for any resulting biases between the samples. As any effect on the intention to use should be positive under the assumption that older adults that fill in their questionnaire online are more likely to adopt mHealth and our findings indicate that the intention to use mHealth has not (or only slightly) increased, this limitation appears to have had no (or little) effect on the controlled results.

Another limitation is that we collected data shortly after the onset of the pandemic in the spring of 2020. This was a turbulent period in which measures restricting the delivery of care were introduced, and in which those same measures were subject to change. However, the greater share of measures the Dutch government took at the start of the COVID pandemic to restrict in-person healthcare delivery have been consistently enforced throughout the data collection period (62). Our results may not be valid for later stages of the pandemic, in which measures were different and knowledge of the disease and fatality rates increased, and (adherence to) restrictive measures weakened. Future research on later stages of the pandemic and the role of such pandemic-related factors on changes in ITU, if any, is called for especially because of the vulnerability of older adults during COVID pandemic conditions.

## Conclusion

The intention of Dutch older adults to use mHealth has not changed during the first months of the pandemic. The extended TAM model has robustly explained intention to use, with only minor differences after the first months of the pandemic. Future studies on later stages of the pandemic are needed to investigate possible long-term effects of the pandemic circumstances on the ITU of the older adult population. Further policy measures to promote uptake of mHealth among Dutch older adults might target facilitation and support for example by health care providers, rather than the support of family and friends. Future research on this topic might consider using a more sensitive model in which intention to use is modeled using a continuous variable, as this may pick up policy effects earlier.

## Data availability statement

The raw data supporting the conclusions of this article will be made available by the authors, without undue reservation.

## Ethics statement

The studies involving human participants were reviewed and approved by Medical Ethical Commission of the Erasmus University of Rotterdam under the number MEC-2018-120. The patients/participants provided their written informed consent to participate in this study.

## Author contributions

MA designed the research project and developed the questionnaire. APN and MA validated the questionnaire. MA and FE collected the data with the help of data assistants. FE performed the analysis under the supervision of MA and JK, and wrote the initial version of the manuscript. All authors interpreted the results and revised the paper critically.

## Conflict of interest

The authors declare that the research was conducted in the absence of any commercial or financial relationships that could be construed as a potential conflict of interest.

## Publisher’s note

All claims expressed in this article are solely those of the authors and do not necessarily represent those of their affiliated organizations, or those of the publisher, the editors and the reviewers. Any product that may be evaluated in this article, or claim that may be made by its manufacturer, is not guaranteed or endorsed by the publisher.

## Abbreviations

OR: Odds ratio
TAM: Technology acceptance model
